# Examining supports and barriers to breastfeeding through a socio-ecological lens: a qualitative study

**DOI:** 10.1186/s13006-021-00401-4

**Published:** 2021-07-12

**Authors:** Kailey Snyder, Emily Hulse, Holly Dingman, Angie Cantrell, Corrine Hanson, Danae Dinkel

**Affiliations:** 1 School of Pharmacy and Health Sciences, Creighton University, Omaha, NE USA; 2 Center for the Child & Community, Children’s Hospital & Medical Center, Lincoln, NE USA; 3 MilkWorks, 10818 Elm St, Omaha, NE USA; 4 Medical Nutrition Education, University of Nebraska Medical Center, Omaha, NE USA; 5 School of Health & Kinesiology, University of Nebraska at Omaha, Omaha, NE USA

**Keywords:** Breastfeeding, Socio-ecological model, Qualitative, Policy

## Abstract

**Background:**

Early breastfeeding cessation is a societal concern given its importance to the health of mother and child. More effective interventions are needed to increase breastfeeding duration. Prior to developing such interventions more research is needed to examine breastfeeding supports and barriers from the perspective of breastfeeding stakeholders. One such framework that can be utilized is the Socio-Ecological Model which stems from Urie Broffenbrenner’s early theoretical frameworks (1973–1979). The purpose of this study was to examine supports and barriers to breastfeeding across environmental systems.

**Methods:**

A total of 49 representatives participated in a telephone interview in Nebraska, USA in 2019. Interviewees represented various levels of the model, based on their current breastfeeding experience (i.e., mother or significant other) or occupation. A direct content analysis was performed as well as a constant comparative analysis to determine differences between level representatives.

**Results:**

At the Individual level, breastfeeding is a valued behavior, however, women are hindered by exhaustion, isolation, and the time commitment of breastfeeding. At the Interpersonal level, social media, peer-to-peer, and family were identified as supports for breastfeeding, however lack of familial support was also identified as a barrier. At the community level, participants were split between identifying cultural acceptance of breastfeeding as support or barrier. At the organizational level, hospitals had supportive breastfeeding friendly policies in place however lacked enough personnel with breastfeeding expertise. At the policy level, breastfeeding legislation is supportive, however, more specific breastfeeding legislation is needed to ensure workplace breastfeeding protections.

**Conclusion:**

Future efforts should target hospital-community partnerships, family-centered education, evidence-based social media strategies and improved breastfeeding legislation to ensure breastfeeding women receive effective support throughout their breastfeeding journey.

## Background

The importance of breastfeeding for child and mother is well-established [1]. Not only can breastfeeding support infant survival in the first year of life, breastfeeding mothers also have a lower risk of type 2 diabetes, hypertension, breast and ovarian cancer [1–3]. Despite the significant health benefits, breastfeeding rates are still well-below the recommended duration of exclusive breastmilk feeding for 6 months followed by continued breastfeeding with the addition of complementary food [1–5]. Global research suggests that concurrent intervention delivery using a combination of systems such as home, family, healthcare and community involvement improves breastfeeding rates; however, few successful interventions are currently in practice [6–9]. One such theory that can be used to understand human development is Urie Brofenbrenner’s bioecological theory [10].

Brofenbrenner’s theoretical perspective has evolved greatly over time, however his early work (1973–1979) provides a strong foundation for understanding the complexities of engaging in a behavior like breastfeeding [11–15]. According to Brofenbrenner, “the ecology of human development involves the scientific study of the progressive, mutual accommodation between an active, growing human being and the changing properties of the immediate settings in which, the developing person lives, as this process is affected by relations between these settings, and by the larger contexts within which the settings are embedded” [16]. Broffenbrenner posited there were four types of systems that could bidirectionally influence development (microsystem, mesosystem, exosystem and macrosystem). Important to this study, the microsystem was defined as the proximal setting where one can have individualized interactions (e.g., home, childcare, work, healthcare) [12]. A breastfeeding women’s microsystem consists of many individuals that have the potential to influence her breastfeeding journey. For instance, her family and/or friends, childcare providers, healthcare providers and public health professionals. Despite the substantial influence these individuals may have on a breastfeeding woman’s experience, few studies have attempted to understand the reciprocal interaction between mothers and individuals within their microsystem. Importantly, a comparison of perspectives of those occupying various microsystems would help elucidate the proximal processes influencing a mother’s breastfeeding journey [15].

Globally, Broffenbrenner’s conceptual framework has been frequently adapted to help elucidate health promotion endeavors [10]. This framework is often identified as the Socio-Ecological Model (SEM) [10]. While the SEM has been utilized in health promotion research there are criticisms of inappropriate use associated with the evolution of Broffenbrenner’s theory over his lifespan [17]. This study will focus primarily on interpreting Brofenbrenner’s early theoretical perspective (1973–1979) [11–15]. The SEM holds that health behaviors are affected by the interaction between an individual, their community and their environment. Typically, spheres of individual, interpersonal, community, organizational and policy environments are considered. The first level, individual, includes attributes such as personal knowledge, attitude and behavior. The second level, interpersonal, includes formal and informal social support systems. This support typically stems from family, friends, peers and co-workers. The third level involves the community level and focuses on how community organizations provide formal and informal support. The fourth level, organization, focuses on rules and regulations that affect how services may be provided to an individual. Finally, the fifth level, policy, focuses on local, state, national and global policies that can influence resource allocation and access [18]. For the purposes of this study, policies will be specific to United States breastfeeding policies. In the United States, all states have laws that allow women to breastfeed in any public or private venue. The United States also requires employers to provide reasonable break time for mothers to express milk for one year after childbirth [8].

Importantly, a women’s breastfeeding journey can be impacted by factors at each level of the Socio-Ecological Model. For example, research has shown factors such as low self-efficacy (individual), lack of partner support (interpersonal), community stigma (community), hospital formula samples (organizational) and lack of protective laws (policy), hinder breastfeeding [19–22]. Conversely, factors at each level have been identified as breastfeeding supports such as high self-efficacy (individual), supportive family and friends (interpersonal), access to community resources (community), in-hospital education (organizational) and workplace protections (policy) [8, 23–25]. Furthermore, there are individuals within a breastfeeding women’s microsystem that can influence each level of the Socio-Ecological Model [17].

To the researchers’ knowledge few studies have utilized SEM to explore breastfeeding behavior [26, 27]. The studies that have been conducted were limited to the perspectives of mothers and healthcare providers. Further research is needed to understand factors across SEM levels to understand how to best support women in their breastfeeding journey. Exploration of the perspectives of individuals who directly interact within a women’s microsystem and represent each level of the SEM is critical to the development of further interventions. To the best of the researchers’ knowledge such a study has not previously been undertaken.

## Methods

A cross-sectional qualitative design guided by the SEM utilizing grounded theory was employed [28]. Participants were recruited via purposive sampling throughout the state of Nebraska in both urban and rural areas [29].

Representatives of each level of the SEM were recruited according to their professional breastfeeding history. Recruitment methods included an e-mail to current members of the State Breastfeeding Coalition and posting interview information on local and statewide *Facebook* breastfeeding support groups. Interested participants were encouraged to contact the first author to arrange a time for an interview. A goal of 12 participants per level was sought, however, once the ongoing data analysis reached saturation within a level, participant recruitment was halted (*n* = 49).

A total of 49 telephonic semi-structured interviews were conducted between the months of May and August 2019. All research procedures were approved by an ethics review board. Participants were informed of the study’s purpose and risks; that their participation was voluntary, and they had the right to withdraw at any time; their details such as age, race/ethnicity and occupation were documented. The duration of each interview was approximately 25 min.

A 15-question semi-structured interview guide based on the SEM was employed. The interviews focused on participant perceptions of how the various levels of the SEM supported or hindered breastfeeding. The questions were developed by a qualitative research expert and piloted with a representative from each of the five SEM levels. Face validity was conducted on the five pilot interviews to ensure the wording was clear and interpreted accurately [30].

A grounded theory framework was utilized to establish the validity of findings [29, 31]. All interviews were transcribed by the interviewer and reviewed for accuracy by the primary author. A content analysis was employed to identify preliminary coding of categories based on operational definitions of SEM constructs. The steps involved included two researchers reviewing all interview transcripts twice. Two researchers separately identified and coded statements that directly related to one of the SEM levels. Both made notes throughout the coding process and resolved discrepancies through discussion [32]. The next step was a constant comparative analysis. This analysis involved both researchers scrutinizing the responses of participants representing each level of SEM to compare their experiences and identify categories of significance. This process facilitated the strategy of intuiting, that is, identification of themes identified in participant’s accounts [33], resulting in a better depth of analysis of the stakeholders’ reflections at each level of Socio-Ecological Model. The resultant set of subthemes were listed under the initial themes identified. A further analysis of rural versus urban participants was also undertaken.

Peer debriefings with a peer without breastfeeding expertise took place throughout the process of analysis in an attempt to reduce analyzer bias [31]. A second qualitative researcher (last author) reviewed the themes identified from the content analysis and comparative analysis. Consensus was achieved through discussion.

## Results

A total of 49 participants were interviewed. Twelve breastfeeding mothers were recruited at the Individual level. Participants at the Interpersonal level offered personal support to breastfeeding mothers. This group included six in-home childcare providers and four partners of breastfeeding mothers. Twelve participants were recruited at the Community level. Six were childcare center directors, one was a peer lactation counselor, three were certified lactation counselors, and the remaining two were a social worker and medical librarian/community advocate respectively. The twelve participants recruited at the Organizational level worked in an administrative capacity and included eight community program administrators/managers and two maternal/child health nonprofit directors. Finally, those at the Policy level included a Health Department Division Chief, a medical doctor and four International Board Certified Lactation Consultants. The mean age of the 49 participants was 38.7 ± 10.1. Most participants resided in an urban residence.

### Supports and barriers to breastfeeding

**Fig. 1:**
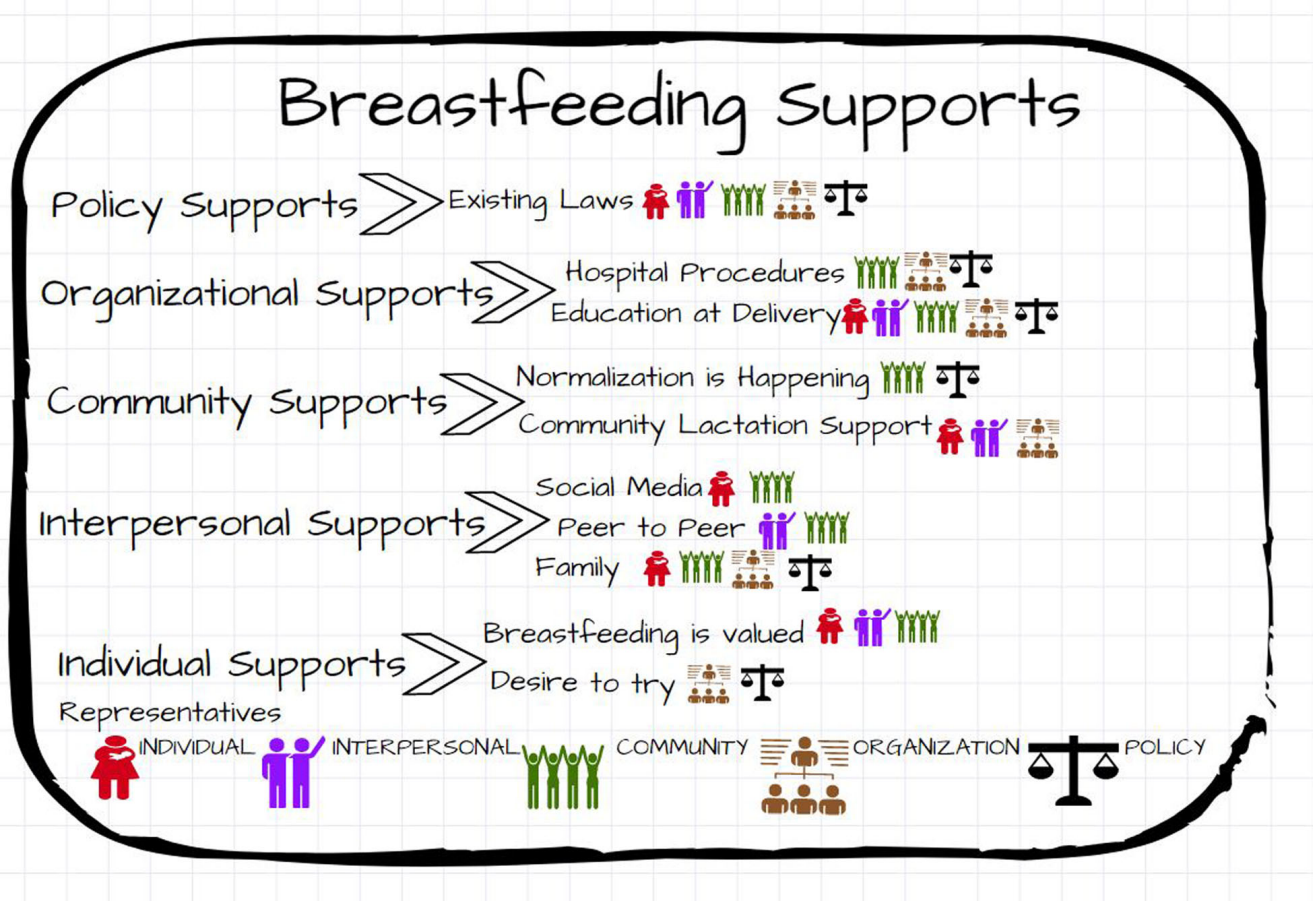
Breastfeeding support themes

**Fig. 2:**
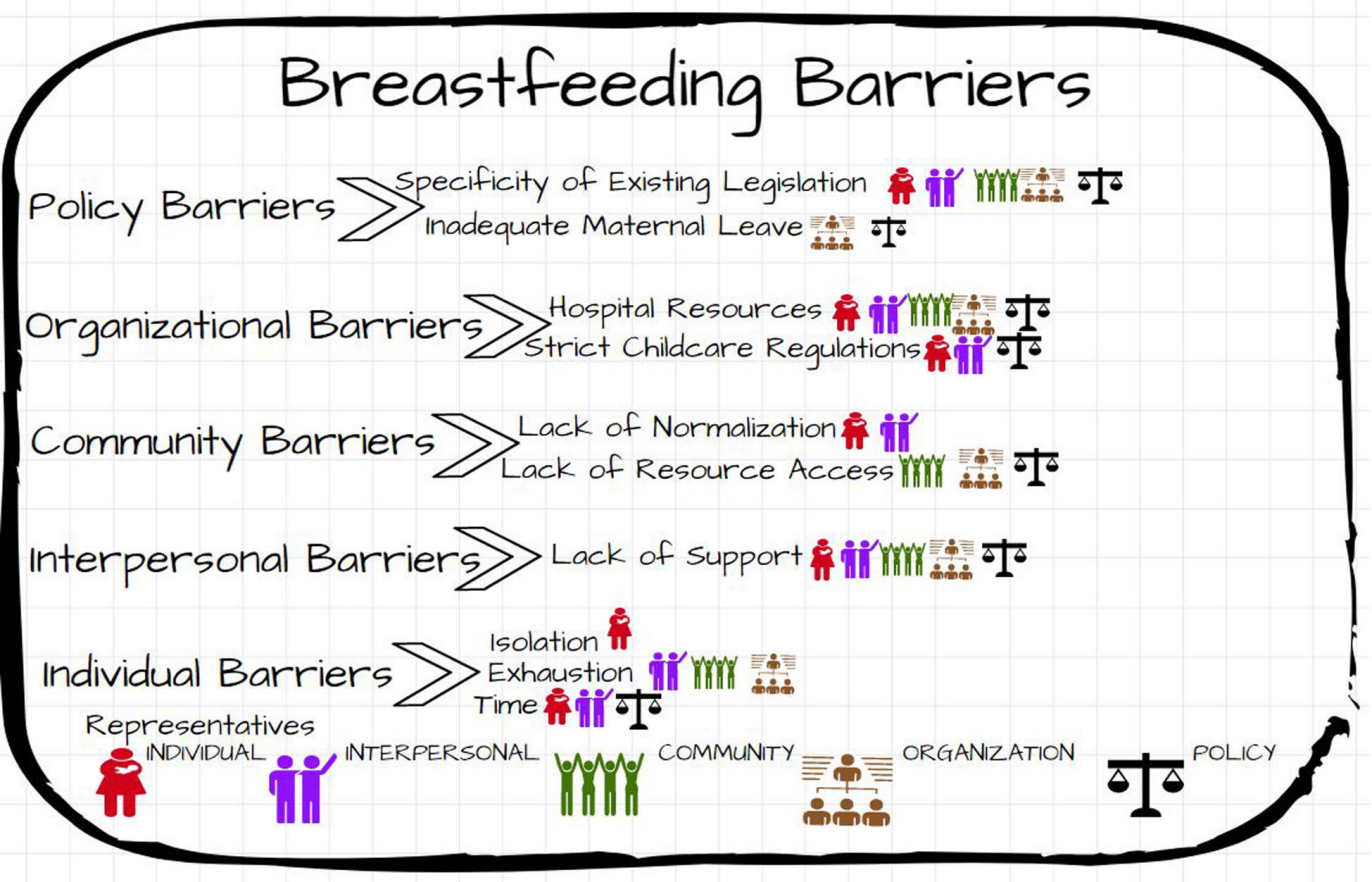
Breastfeeding barrier themes

Figures 1 and 2 list the major themes identified at each level of the Socio-Ecological Model. The stakeholders identifying each theme are denoted via a symbol. Figure 1 notes the most reported themes related to breastfeeding support identified from the interviews. Figure 2 identifies the most common barriers to breastfeeding.

### Individual factors

At the Individual level, two main themes related to breastfeeding support were identified, namely, breastfeeding is a valued behavior; and mothers' desire to try to breastfeed. For instance, a Community Health coordinator reported:

*“I think it is becoming more popular nowadays, at least [to] attempt to start breastfeeding. Women will brag that they made it [for] a whole year or breastfed [for] six months.”*

Individual barriers to breastfeeding typically involved time commitments, exhaustion and isolation. The time commitment was the most frequently reported issue. For example, a labor and delivery nurse stated*, “....just the time commitment of it. I mean I always say it’s not hard, it’s just demanding. You [have] to live on a two-hour clock*.” Exhaustion was also a common theme. For instance, a Community Program Coordinator observed*, “I think the lack of sleep that comes with a newborn....You know, you’re not well-rested and you’re trying to have good mental health and it’s a struggle*.” Additionally, breastfeeding mothers reported isolation as an issue, with one mother admitting, “*I would definitely say like, the kind of the isolation factor of it. You’re the only one who can do it and sometimes it’s a little lonely; just feeling stuck sometimes”*.

### Interpersonal factors

At the Interpersonal level, social media, peer-to-peer contact, and family were identified as the greatest sources of support. For example, a County Health Director observed, *“I see a really strong social media presence, a supportive social media presence. It seems like women are going to social media to find support.”* Further, a husband of a breastfeeding women noted, “*I think what really helped my wife was the support groups she found that allowed for mother-to mother peer counseling.”* Finally, familial support was often recognized as a key influencer of breastfeeding support. One community program coordinator indicated, “*Some of the biggest support pieces that I feel like are critical, are having support from your own family.”*

The main barrier identified was a lack of support from family and/or friends. For example, one social worker stated: *“A lot of our moms, want to breastfeed, and they don’t have a lot of support from like dads or friends.”*

### Community factors

Several interviewees reported feeling that breastfeeding out in the community was becoming more culturally accepted. A childcare provider observed, “*I think it’s becoming better, it’s more socially normally to see a mother breastfeeding in public. I think it’s not as shunned upon not to [breastfeed] in public and everything.”* The existence and availability of community lactation support was also identified as a breastfeeding support. Many participants reported their awareness of several community organizations or support groups. For example, one breastfeeding mother reported, “*definitely places like [community breastfeeding non-profit] for lactation support... it’s helpful. I feel like [it is helpful to] just to have places like that in the community that women can go [to].”*

A lack of community resources in rural and under-served areas was identified as a community-level barrier. One nurse residing in a rural area reported, “*we have very minimal support. When I moved here, I searched for support groups and there was nothing to be found*.” Despite many reporting a cultural acceptance of breastfeeding, others did report a lack of breastfeeding acceptance. One mother observing, “*It’s just hard to breastfeed in public. I know it’s supposed to be a thing you can do everywhere but sometimes it’s just not really looked at as acceptable yet”.*

### Organization factors

At the Organizational level, support was associated with hospitals having helpful policies in place regarding breastfeeding and effective in-hospital education directly after birth. For example, a home visiting International Board Certified Lactation Consultant (IBCLC) stated,

“*I think they [hospitals] have done a great job with all of the new policies that we’ve put in place so, the sacred hour, skin-to-skin, delaying the bath, they’ve put a lot of things in place to help breastfeeding moms*.”

Conversely, although not a majority, two healthcare providers stated they worked in facilities where mothers were distributed formula even prior to their child’s birth. For example, a labor and delivery nurse residing in a rural area pointed out,

“*They give out formula at your first visit when you come to the hospital to register before you come in for delivery . . . they send you home with a bunch of [formula brands].”*

A lack of hospital personnel despite good procedures was also cited. For example, one IBCLC observed,

*“It would be nice if they could have more CLCs [certified lactation counselor] or IBCLCs on the staff because what I hear from families is that there was an IBCLC there, but they weren’t able to spend much time with them”.*

### Policy factors

At the Policy level, current law and workplace protections were reported by most participants. One IBCLC stated, *“I think they [the laws] have been very helpful, especially with moms going back to work, you know, the law [that allows a woman to breastfeeding public] and the pumping laws have definitely been a huge help”.*

Conversely, most participants felt there remained a lack of specificity within existing United States breastfeeding laws/policies that left women unprotected. One community program manager noted,

*“I know there are policies and laws, but I feel like some of those still have loopholes. Like it doesn’t seem to cover every occupation, especially those teachers and nurses who need varying pumping schedules.”*

## Discussion

This qualitative inquiry undertook a unique investigative approach utilizing SEM and interviewing individuals within a breastfeeding women’s microsystem to understand their breastfeeding supports and barriers [11–15]. We have identified areas of improvement that should be considered based on the findings of this study.

Several breastfeeding supports were identified that appear to influence breastfeeding initiation. For example, at the Individual level, mothers have a desire to try to breastfeed and breastfeeding is seen as valuable. Additionally, at the Organizational level, hospitals have breastfeeding friendly policies in place immediately after childbirth. Desiring to try to breastfeed, valuing breastfeeding and having supportive hospital breastfeeding policies align with the high breastfeeding initiation rates (84.7%) seen in the United States [34]. However, there appears to be a disconnect regarding support for breastfeeding upon hospital discharge. This study identified barriers such as the time commitment of breastfeeding, maternal exhaustion/isolation, lack of familial support, lack of cultural acceptance and lack of specificity in workplace protections.

There are multiple ways to reduce the barriers that may be contributing to early breastfeeding cessation upon hospital discharge. Importantly, there is a large drop in exclusive breastfeeding that occurs within the first two weeks postpartum [34]. One way to combat this early decline in breastfeeding could be to develop opportunities to bridge the gap between hospital and community breastfeeding support organizations. For example, hospitals could use community lactation providers to support in-hospital education classes or even support protocols such as telephone follow-up. Further, healthcare workers in the hospital setting should be aware of all the community resources available (e.g., La Leche League, community *Facebook* groups, community non-profit organizations) and be able to effectively refer women to these resources.

Further, although peer to peer and familial support for breastfeeding was identified, familial support for breastfeeding remained the largest barrier identified at the Interpersonal level. Family centered breastfeeding education should be considered and ideally should occur prior to childbirth. Inclusion of significant others and extended family members (i.e., grandparents) in prenatal breastfeeding education has been associated with longer breastfeeding durations [19]. Inclusion of family members in breastfeeding education may also help reduce the feelings of isolation and exhaustion reported by breastfeeding mothers. Healthcare providers should consider inviting extended family members to prenatal appointments and early infant well-checks to receive breastfeeding education.

Cultural acceptance of breastfeeding was identified as a breastfeeding support at the Community and Organizational levels, conversely, a lack of cultural acceptance for breastfeeding was reported at the Individual and Interpersonal levels. This discrepancy in perceptions of cultural acceptance of breastfeeding suggests that despite communities and organizations having policies and procedures that are supportive of breastfeeding, breastfeeding women still feel stigmatized. Increasing the frequency and amount of breastfeeding information available to non-breastfeeding individuals is crucial to enhancing cultural acceptance. One outlet that can be utilized as a platform for providing breastfeeding information is social media (i.e., *Instagram, Facebook*). Social media is often utilized to provide education and peer to peer support to breastfeeding women, however, it is underutilized as a tool to provide breastfeeding information to non-breastfeeding individuals [35–37]. Healthcare professionals/agencies could consider using social media (i.e., *Instagram/Facebook* posts) to target breastfeeding education towards non-breastfeeding individuals to increase cultural acceptance of breastfeeding.

Finally, while substantial improvements in breastfeeding legislation have occurred over the past 20 years, the lack of specificity of these laws is putting a mother’s breastfeeding journey at risk. Despite United States’ legislation requiring “reasonable” time for pumping breaks during the workday, women are experiencing barriers such as lack of schedule control, unequal access to space and unexpected breastfeeding demands [38]. Our study found participants across SEM levels identified the need for more specific workplace protections. While more specific legislation is pertinent, research also shows the most common facilitators of workplace breastfeeding are access to space and coworker support. Workplace policies could be designed to ensure space, and employer education could be conducted to enhance workplace acceptance of breastfeeding [39]. More importantly, increasing the length of maternity leave within the United States to 6 months would reduce workplace pumping associated barriers and increase the prevalence of exclusive breastfeeding [40].

### Limitations

A weakness of this study was that it was limited to only one state in the Midwestern United States, further research is needed in other geographic locations to determine the generalizability of findings.

## Conclusion

Representatives at each level of the SEM have identified key supports and barriers to breastfeeding. At the Individual level, breastfeeding is a valued behavior and women have a desire to try to breastfeed. However, women are hindered by the time commitment of breastfeeding, exhaustion, and isolation. At the Interpersonal level, social media, peer-to-peer, and family were identified as supports for breastfeeding, however, lack of familial support was also identified as a barrier. At the Community level, participants were split between identifying cultural acceptance of breastfeeding as support or a barrier. At the Organizational level, hospitals had supportive breastfeeding friendly policies in place but lacked enough personnel with breastfeeding expertise. Finally, at the Policy level, while supportive breastfeeding legislation exists, it lacks the specificity needed to offer workplace protections for all breastfeeding women in the United States. Future research and intervention efforts should focus on bridging hospital and community partnerships, increasing family centered breastfeeding education, enhancing evidence-based social media strategies, and increasing the specificity of workplace breastfeeding protections which include longer maternity leaves.

## Data Availability

The datasets used and/or analyzed during the current study are available from the corresponding author on reasonable request.

## Abbreviations

CLC: Certified Lactation Counselor
IBCLC: International Board Certified Lactation Consultant
SEM: Socio-ecological model
